# Temporal refuges of a subordinate carnivore vary across rural–urban gradient

**DOI:** 10.1002/ece3.9310

**Published:** 2022-09-21

**Authors:** Rumaan Malhotra, Samantha Lima, Nyeema C. Harris

**Affiliations:** ^1^ Ecology and Evolutionary Biology University of Michigan Ann Arbor Michigan USA; ^2^ Forestry and Natural Resources Purdue University West Lafayette Indiana USA; ^3^ Applied Wildlife Ecology Lab School of the Environment, Yale University New Haven Connecticut USA

**Keywords:** coyote, landscape of fear, Michigan, niche, partitioning, raccoon

## Abstract

Animals exhibit variation in their space and time use across an urban–rural gradient. As the top‐down influences of apex predators wane due to human‐driven declines, landscape‐level anthropogenic pressures are rising. Human impacts can be analogous to apex predators in that humans can drive increased mortality in both prey species and carnivores, and impact communities through indirect fear effects and food subsidies. Here, we evaluate the time use of a common mesocarnivore across an urban–rural gradient and test whether it is influenced by the intensity of the use of a larger carnivore. Using multiple camera‐trap surveys, we compared the temporal response of a small carnivore, the raccoon (*Procyon lotor*), to the larger coyote (*Canis latrans*) in four study areas across Michigan that represented a gradient of pressure from humans. We found that raccoon time use varied by study area and was most unique at the rural extreme. Raccoons consistently did not shift their activity pattern in response to coyotes in the study area with the highest anthropogenic pressures despite the considerable interannual variation, and instead showed stronger responses to coyotes in more rural study areas. Temporal shifts were characterized by raccoons being more diurnal in areas of high coyote activity. We conclude that raccoons may shift time use in the presence of coyotes, dependent on the level of anthropogenic pressure. Our results highlight that the variation in raccoon time use across the entirety of the urban–rural gradient needed to be considered, as anthropogenic pressures may dominate and obscure the dynamics of this interaction.

## Graphical abstract

Anthropogenic forces can alter temporal interactions between species. We compared the time use of a small carnivore across an urban‐rural gradient, and tested whether it responded to the presence of a larger carnivore. We found that both time use and the response to a larger carnivore varied along the gradient, especially at the extremes.

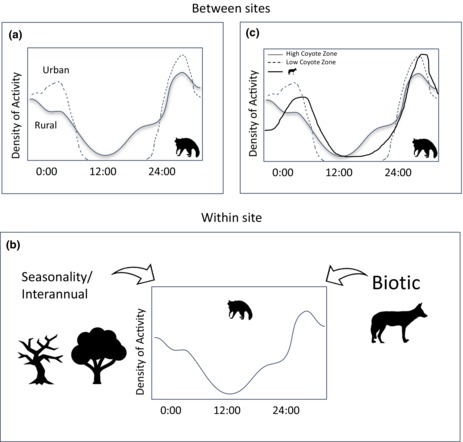

## 1 INTRODUCTION

Urban–rural gradients provide comparisons between natural (e.g., top‐down predation) and anthropogenic forces (e.g., fear of humans) that structure wildlife communities through behavioral and ecological pathways (Ellington & Gehrt, 2019; McDonnell & Pickett, 1990). Non‐consumptive effects that apex predators exert on prey or smaller competitors commonly manifest as antipredator behaviors (Wirsing et al., 2021). Similar to apex predators, humans can induce non‐consumptive effects on subordinate species through changes in space and time use (Ciuti et al., 2012; Clinchy et al., 2016). However, humans are unique in their top‐down pressures in that they can exert fear effects across trophic levels, superseding hierarchies in natural systems (Smith et al., 2017; Suraci et al., 2019). Thus far, urban–rural gradients have predominantly highlighted changes in diet and physical characteristics (e.g., body size) that can affect ecological interactions, or changes in biodiversity and species composition across taxa (Gámez et al., 2022; Marzluff, 2001; Urban et al., 2006). Although not specifically cast in an urban–rural framework, there is further evidence that humans and built structures can alter animal behavior (Avilés‐Rodríguez & Kolbe, 2019; Van Donselaar et al., 2018). For example, global meta‐analyses found that the intensity of human pressure can drive increased nocturnality and reduce movement (Gaynor et al., 2018; Tucker et al., 2018). Altered time use due to humans can further translate into altered interspecific interactions, for example, by increasing the total spatiotemporal overlap and thus the probability of encounter (Gallo et al., 2019; Lewis et al., 2015). In urban areas, where spatial overlap among species is inevitable due to the limited amount of habitat available, temporal partitioning may be particularly important for species' persistence (Adams & Thibault, 2006; Santos et al., 2019; Stark et al., 2020). We leverage an urban–rural gradient formed by human pressure to examine spatiotemporal dynamics between a widely distributed carnivore and a smaller sympatric competitor.

As a highly adaptive mesocarnivore, coyotes (*Canis latrans*) exploit a wide range of habitats and exhibit tolerance to disturbance (Bekoff & Gese, 2003; Flores‐Morales et al., 2019). Coyotes exemplify mesopredator release, a phenomenon in which subordinate carnivores increase in abundance and distribution once the suppressive effects of larger carnivores are removed (Crooks & Soulé, 1999; Prugh et al., 2009). For example, the recent range expansion of coyotes aligns with the human‐caused extirpation of gray wolves (*Canis lupus*). Though coyotes are subordinate to larger carnivores where they are sympatric, they are aggressors toward several smaller carnivores and account for high rates of mortality for some species (e.g., *Vulpes velox* and *Vulpes macrotis*) (Bekoff & Gese, 2003; Berger, 2007). As a result, coyotes are commonly cited as a species that can act as both a mesopredator or an apex predator in their community, depending on the presence of larger carnivores such as the gray wolf (Colborn et al., 2020; Prugh et al., 2009; Roemer et al., 2009) or mountain lion (*Puma concolor*) (Elbroch & Kusler, 2018; Ruprecht et al., 2021). Similarly, raccoons (*Procyon lotor*) exhibit tolerance to human pressures and spatially overlap with coyotes through much of their North American range (Timm et al., 2017). Coyote‐raccoon interactions are interesting because both species are widespread, and exhibit a size difference that should typify intraguild aggression or predation (Donadio & Buskirk, 2006). Despite this, we lack evidence for any sort of spatial or temporal partitioning between coyotes and raccoons (Gehrt & Clark, 2003; Shedden et al., 2020). There has yet to be a study that examines the temporal dynamics of these two species across the urban–rural gradient.

Raccoons exhibit spatiotemporal variation in behavioral attributes, leading us to the expectation that the response of raccoons to coyotes may vary by habitat and other characteristics across study areas (Beasley et al., 2011). Based on a lack of avoidance behavior by raccoons or raccoon mortality due to coyotes, Gehrt and Prange (2007) argued that raccoons and coyotes do not fit into the mesopredator release hypothesis. There is little evidence that coyotes act as a control on the abundance or spatial use of raccoons (Lesmeister et al., 2015). Telemetry studies of raccoons have found some evidence of mortality due to coyotes, but only as a rare occurrence (Gehrt & Clark, 2003; Prange et al., 2003). In North Carolina, raccoons exhibited low levels of vigilance despite their temporal overlap with coyotes (Chitwood et al., 2020).

**FIGURE 1:**
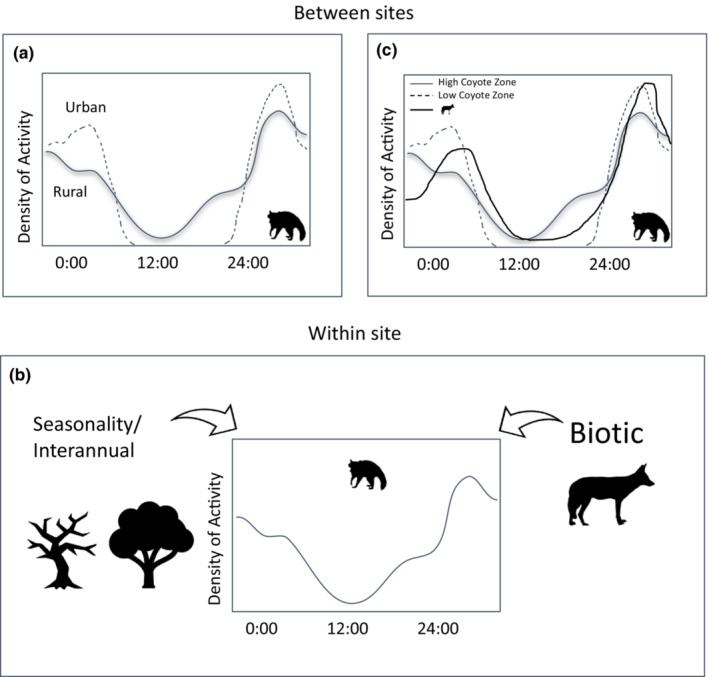
The three comparisons considered within our study: (a) raccoon temporal activity was compared between study areas; (b) raccoon temporal activity was compared between years, and across zones of coyote intensity of use within each study area; and (c) raccoon temporal activity results from the within study area comparisons in response to coyotes were compared across study areas.

Given that coyotes pose some risk to raccoons based on size and sympatry, but that overall risk is low, we tested whether raccoons shifted their activity based on heterogeneity in coyote risk within a study area. Employing a camera survey across an urban–rural gradient, we tested whether raccoon time use differed between intensities of coyote spatial use. Specifically, we tested the variation in raccoon and coyote time use across two scales: between study areas (across the urban–rural gradient) and within the study area, between years (interannual variation) (Figure 1). As anthropogenic pressures increase, our knowledge of contemporary baseline ecological interactions becomes dated. Thus, it becomes essential to understand how these competitive interactions compare across landscapes with varying human pressures. Therefore, we tested three hypotheses based on wildlife prioritizing the avoidance of human activity in their temporal activity:

1. Raccoon time use in the most urban study area will significantly differ from the other three study areas.
2. Interannual variation in both raccoon and coyote activity will be lowest in the urban study area.
3. Raccoon time use will shift in areas of high coyote activity at the rural end of the urban–rural gradient, but not at the urban end.

## 2 METHODS

### 2.1 Study area

**FIGURE 2:**
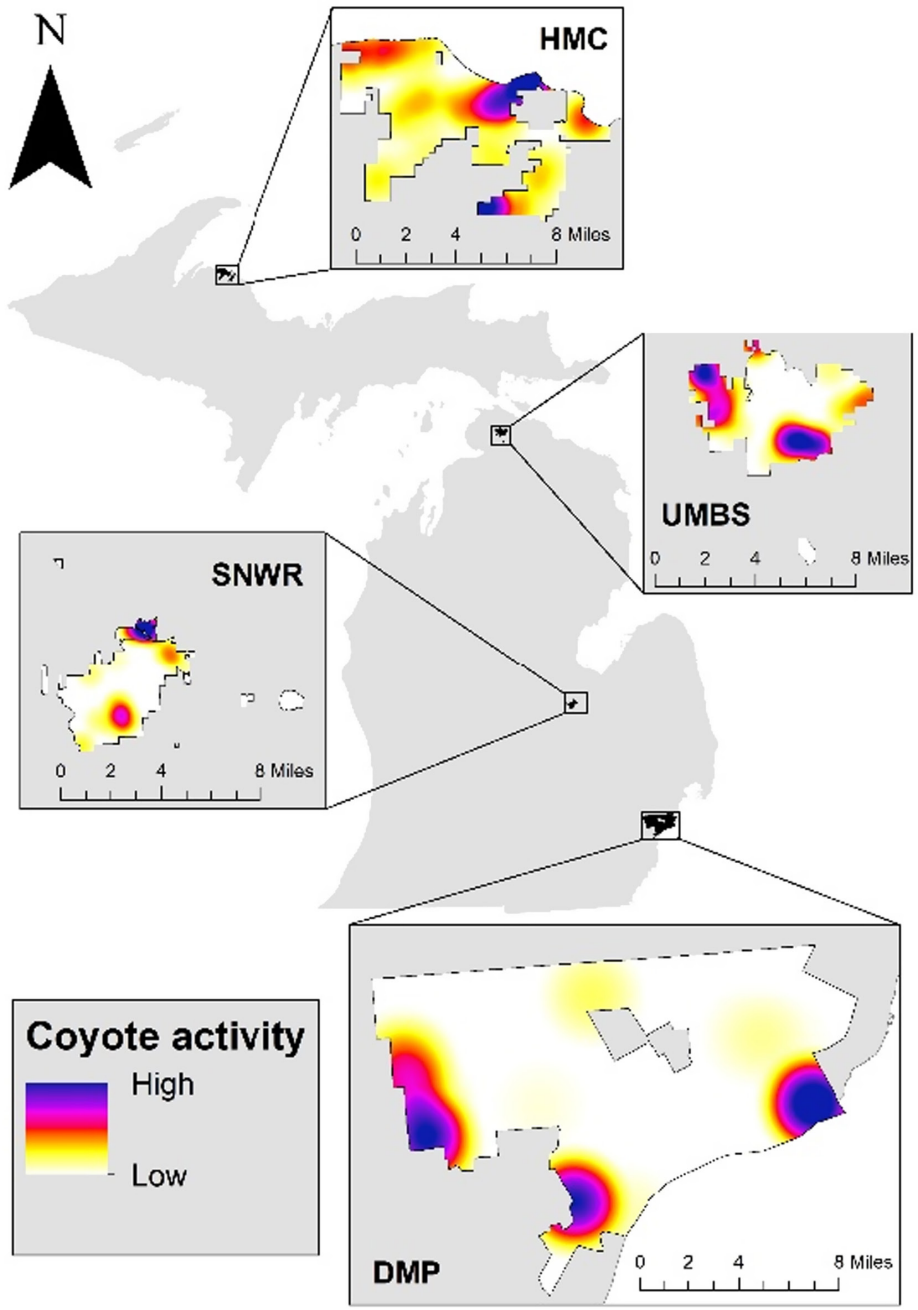
Study sites across Michigan. From north to south, the Huron Mountain Club (HMC), the University of Michigan Biological Station (UMBS), the Shiawassee National Wildlife Refuge (SNWR), and the Detroit Metroparks (DMP). Example coyote spatial activity kernel density hotspots are included for each site; hotspots in coyote detections varied by year, and KD maps were generated for each survey.

We investigated raccoon temporal dynamics across differing levels of coyote activity in four study areas across the state of Michigan, USA (Figure 2) which represent an urban–rural gradient.

1. The Huron Mountain Club (HMC) is a privately‐owned property along the southern shore of Lake Superior, encompassing around 6900 hectares in Marquette County, Michigan, USA. This study area has a wide variety of habitats including beech‐sugar maple hardwood forests, aspen‐dominated stands, and coniferous boreal forests. Sympatric large predators include: gray wolves, black bears (*Ursus americanus*), and coyotes. Anthropogenic pressures are limited to a small, seasonally occupied area of human habitation near the north‐central part of the property. Hunting and fishing occur on the property, and the intensity is presumably low due to restrictive public access.
2. The University of Michigan Biological Station (UMBS), a ~4000‐hectare research station and forest in Pellston County, Michigan, USA served as one of our intermediate disturbance study areas. With repeated logging and fire disturbance until 1923, the secondary forest is a mix of transitional hardwood and boreal forests. Douglas and Burt lakes along the north and south, and the town of Pellston and a major highway along the west and east, respectively, border this study area. Large co‐occurring predators include: black bears, coyotes, and coyote‐wolf hybrids. We were able to distinguish the few known coyote‐wolf hybrids in the area due to them having collars from a different study, which were visible in the camera trap images (Wheeldon et al., 2012). Human pressures resulted from regulated research infrastructures for climate monitoring and housing facilities with low levels concentrated seasonally during the summer.
3. The Shiawassee National Wildlife Refuge (SNWR) is a 9870‐hectare wildlife refuge managed by the US Fish and Wildlife Service. The refuge is comprised of forested hardwood wetlands and prairie. The city of Saginaw abuts the northern edge of the refuge and is surrounded by agricultural land for crop farming. The only large native predator present is the coyote. Anthropogenic pressures, in addition to the urban and ex‐urban nature of the boundaries, are in the form of recreational visitors and hunting. Public hunting for deer and waterfowl, and furbearer trapping are permissible on the refuge in accordance with lawful seasons.
4. The Detroit Metro Parks (DMP) is a noncontiguous collection of greenspaces interspersed throughout southeast Michigan that is managed by the Detroit Parks and Recreation Department. We chose 25 of these parks that varied in size from ~1.6 to 480 hectares, tree cover, human visitation, and degree of disturbance. Roads, buildings, or a riverine edge bound all parks. The only large native predator present is the coyote. Strong anthropogenic pressures are present in the form of the surrounding urban matrix as well as the associated presence of humans and domestic pets across parks.

### 2.2 Camera trap survey

We deployed remotely‐triggered camera traps (Reconyx© PC 850, 850C, 900, 900C) throughout each study area with camera placement and sampling design proportional to study area size (Table 1). Our study uses data from three surveys at DMP (2017, 2018, 2020), three surveys at SNWR (2016, 2017, 2018), two surveys at UMBS (2015, 2016), and four surveys at HMC (2016, 2017, 2018, and 2019). Unbaited camera traps were affixed to trees >0.5 m diameter and placed 0.5–1.0 m off the ground. Study area‐specific placement of camera traps was determined by signs of animal activity such as game trails and scat. Camera trap settings included: high sensitivity, the 1‐s lapse between three pictures in a trigger, and a 15‐s quiet period between triggers.

**TABLE 1:** Temporal activity between study areas using Mardia‐Watson‐wheeler test for raccoons (a) and coyotes (b).

Site comparison	*W*	*Df*	*p* value	Δ_overlap_	Δ (CI)
(a) Raccoon					
HMC vs. UMBS	50.001	2	**<.001**	0.791	0.75–0.83
HMC vs. SNWR	40.358	2	**<.001**	0.836	0.79–0.87
HMC vs. DMP	80.085	2	**<.001**	0.764	0.72–0.80
UMBS vs. SNWR	9.654	2	**.008**	0.903	0.88–0.93
UMBS vs. DMP	9.54	2	**.008**	0.937	0.91–0.96
SNWR vs. DMP	63.218	2	**<.001**	0.883	0.87–0.90
(b) Coyote					
HMC vs. UMBS	29.793	2	**<.001**	0.781	0.71–0.85
HMC vs. SNWR	17.087	2	**<.001**	0.830	0.77–0.88
HMC vs. DMP	1.771	2	.412	0.938	0.90–0.97
UMBS vs. SNWR	4.679	2	.096	0.898	0.84–0.94
UMBS vs. DMP	22.872	2	**<.001**	0.815	0.75–0.88
SNWR vs. DMP	10.963	2	**.004**	0.849	0.79–0.90

*Note*: *W* is the test statistic (approximately chi‐sq distributed), and associated degrees of freedom and *p* value are included. Temporal overlap (Δ) coefficients and 95% confidence intervals are also included to assess the overlap in activity patterns between study areas. Temporal activity at each study area was based off the aggregated triggers for all surveys with that study area.

Values were bolded to highlight *p* values that were below the .05 threshold typically used for significance.

Image identifications were initially crowd‐sourced and filtered for carnivores using a public‐science program called *Michigan ZoomIN* in combination with a consensus algorithm and expert validation (Gadsden et al., 2021). Carnivore species identifications were also sorted and confirmed by at least two independent researchers in the Applied Wildlife Ecology Lab.

### 2.3 Temporal activity

Time stamps associated with the camera trap images were used to conduct temporal analyses. Prior to all analyses, a 30‐min delay between triggers was introduced for every species to account for pseudoreplication, given the tendency of some animals to remain in front of the camera trap and trigger it multiple times. Since surveys were conducted across different times of the year, we scaled times to sunrise and sunset times using the *sunTimes* function in the ‘circular’ package in R (Agostinelli & Lund, 2017).

#### 2.3.1 Variation between study areas

For each study species, we first compiled all triggers from each survey within a study area to have an aggregate across years of overall temporal activity at each study area. We then compared raccoon and coyote temporal activity between study areas (Figure 1a) using the Mardia–Watson–Wheeler (MWW) test, which is a nonparametric test of differences in the angular means between samples of circular data using the ‘circular’ package in R (version 4.1.0). When the *W* value is high, it results in a significant *p* value (*p* < .05), which we concluded to mean that the compared temporal activities were different.

#### 2.3.2 Seasonal and yearly variation

Our multi‐site camera study allowed us to compare differences in the temporal activity of our study species based on landscape‐level differences along an urban–rural gradient. Comparing between seasons can confound inferences from the analyses, due to different seasons potentially resulting in different detection rates (Marcus Rowcliffe et al., 2011). While we did not have identical seasonal coverage for every study area, the multiple surveys at every study area resulted in coverage for the entire year of every study area with the exception of UMBS (Figure S1). To determine if there was consistency in study areas regardless of season and year, we compared the temporal activity of each of our study species between each survey within each study area, and then looked for broader patterns across study areas (Figure 1b).

#### 2.3.3 Coyotes on raccoon temporal activity

For each survey, we used a kernel density estimation for the independent coyote triggers and designated the cameras that fell within the top quartile as ‘HIGH’ coyote intensity of use zones in ArcGIS Pro (version 2.3.1). We used this rather than a fixed cutoff value of expected detection rate because our study areas spanned the entirety of the urban–rural gradient and expected detection rates for coyotes vary depending on the composition of a study area (Magle et al., 2014). Coyote triggers were checked for spatial independence using Moran's I prior to kernel density estimation. We compared raccoon temporal activity between the high coyote cameras and the rest of the study area using the MWW test (Figure 1b,c). For additional evidence that temporal shifts by raccoons were due to avoidance of coyotes, we then compared the overlap between coyote and raccoon time use in the two raccoon test groups from the MWW test. To do this, we calculated an overlap (Δ) coefficient of temporal activity for coyotes and raccoons within each group (‘HIGH’ and ‘LOW’ coyote intensity of use) along with 95% confidence intervals generated from 10,000 parametric bootstraps of the temporal distribution models. Δ values range from 0 to 1, with 0 indicating completely distinct and non‐overlapping temporal activity between comparison groups, and 1 indicating complete overlap. Δ_1_ was used for comparisons when one of the sample groups had less than 50 triggers; otherwise, Δ_4_ was used to estimate temporal overlap (Ridout & Linkie, 2009). Finally, the activity distributions were visually assessed to determine qualitative characteristics of shifts (e.g., raccoons shifting toward increased nocturnality in high coyote zones).

## 3 RESULTS

We obtained 1378 coyote and 11,136 raccoon triggers with a 30‐min quiet period across 12 surveys in 82,595 trap nights (HMC—36,868; UMBS—12,953; SNWR—12,477; and DMP—20,297) from 2015 to 2020. Raccoons and coyotes were the most common carnivores in almost every survey, comprising 57–98% of all the carnivore triggers. In Detroit, where domestic dogs (*Canis familiaris*) and cats (*Felis catus*) comprised 35% of the triggers, coyotes were the fourth most common carnivore species after raccoons, cats, and dogs.

### 3.1 Coyote intensity of use

We found no evidence of spatial autocorrelation in coyote detections based on non‐significance in Moran’s I results across all surveys. Kernel density estimates indicated coyotes were distributed non‐randomly in space (Figure 2). At DMP with heavy anthropogenic pressure (average 77 coyote triggers per camera in ‘HIGH’ coyote zones), coyote spatial activity was concentrated in two heavily forested parks and had few human triggers compared to the rest of the surveyed parks in Detroit. In contrast, at HMC which had the least amount of anthropogenic pressure and the presence of wolves, the highest coyote spatial activity occurred in a recreation area that contained several buildings and homes but had few overall triggers (average three coyote triggers per camera in ‘HIGH’ coyote zones). Coyote spatial activity formed distinct zones in SNWR and UMBS as well, and the location of hotspots varied by the survey. Hotspots in these two study areas were not associated with any discernible landscape‐level measures of anthropogenic pressures. Raccoon triggers were recorded within both the low and high zones of coyote spatial activity across all study areas.

### 3.2 Variation in temporal activity between study areas

**FIGURE 3:**
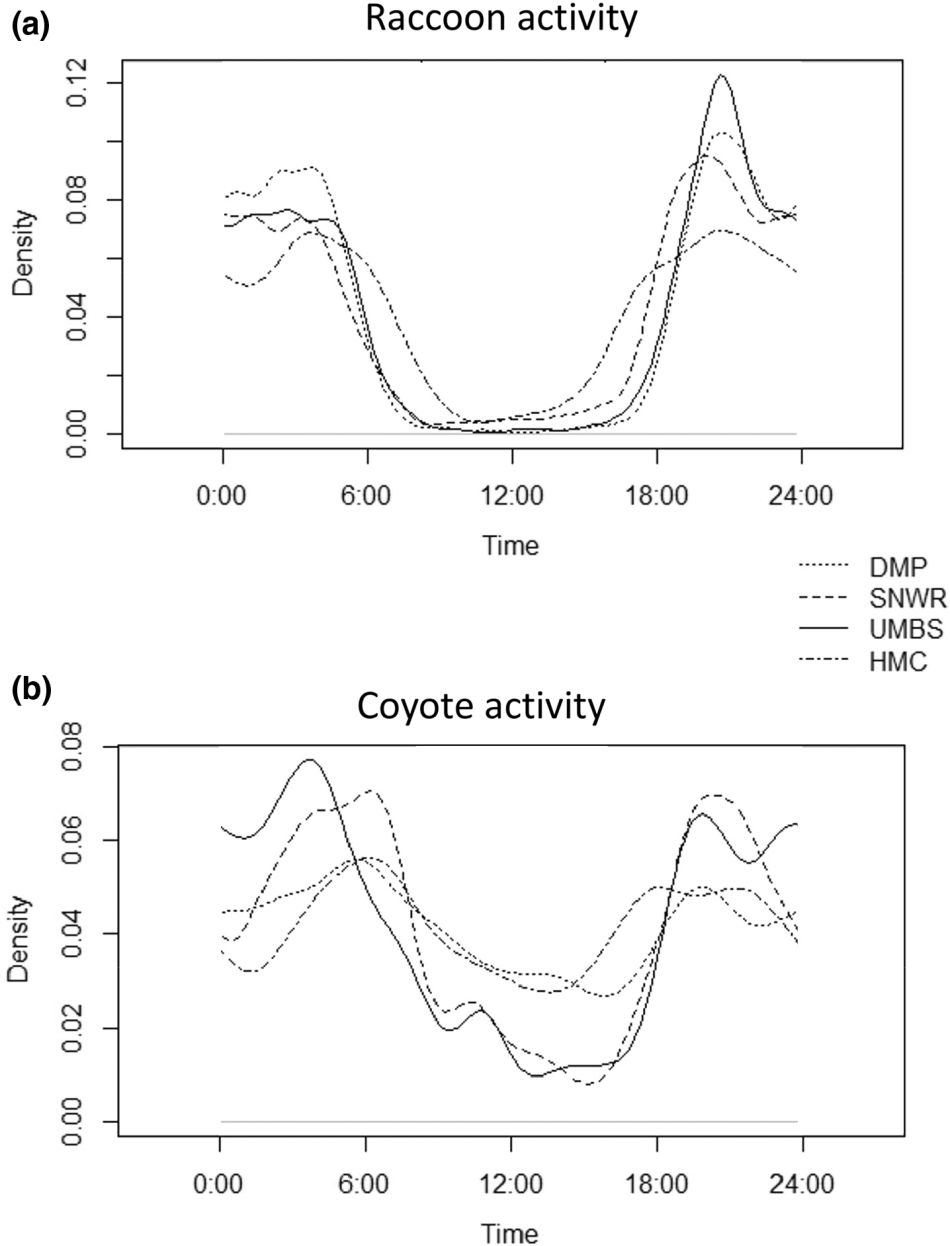
(a) Raccoon activity across all four study areas. Time use of raccoons was summed for all surveys within a study area; (b) coyote activity across all four study areas. Time use of coyotes was summed for all surveys within a study area.

Raccoon activity in each study area was unique, showing significant differences in every pairwise comparison of study areas from MWW tests (Table 1a). We expected raccoon activity to be the most distinct in the most urban study area, DMP. Instead, we found that raccoon activity was most unique at HMC (the opposite end of the gradient), showing considerably more use of the diurnal period (Figure 3a). Overlap comparisons between the sites reflected this. Overlap in raccoon temporal activity between HMC and the other three study areas was relatively low, with confidence intervals for these comparisons showing 76–87% overlap. In contrast, comparisons between UMBS, SNWR, and DMP were significantly higher, with confidence intervals for comparisons between these sites showing 87–96% overlap.

Coyote activity showed a markedly different pattern than raccoon activity did across study areas (Table 1c, Figure 3b). Coyote time use at DMP (the most urban study area) and at HMC (the most rural study area) showed significantly higher overlap than any other area comparisons, which was marked by increased diurnal activity. Coyote time use in the intermediate study areas (SNWR and UMBS) also showed high overlap. Overall, coyote time use fell into two distinct groups: one reflecting the extremes of the gradient and another reflecting the intermediate.

### 3.3 Seasonal/annual variation in temporal activity

Raccoon activity varied significantly by survey and year for every survey on the more urban side of the gradient (SNWR and DMP), but not on the rural end of the spectrum (UMBS and HMC) (Table 2a). At the most urban end of the urban–rural gradient, raccoon activity was significantly different between every year surveyed at DMP and SNWR. At UMBS, the comparison between the 2 years approached significance (*W* = 5.53, *p* = .063). For HMC, the results varied, depending on the years compared. For example, 2016/2017 and 2017/2018 comparisons showed that raccoon time use varied significantly between these years, while raccoon time use between 2018 and 2019 was similar (*W* = 3.03, *p* = .220). These results refuted our hypothesis that interannual variation would be weakest for raccoons at DMP, instead showing that interannual variation is stronger at the urban end of the gradient.

**TABLE 2:** Mardia‐Watson‐wheeler test results comparing temporal activity for raccoons (a) and coyotes (b) at each study area between each survey year

Years	Site	*W*	*Df*	*p* value
(a) Raccoon				
2019 vs. 2018	HMC	3.030	2	.220
2019 vs. 2017	HMC	5.826	2	.054
2019 vs. 2016	HMC	0.228	2	.892
2018 vs. 2017	HMC	22.99	2	**<.001**
2018 vs. 2016	HMC	1.927	2	.381
2017 vs. 2016	HMC	6.77	2	**.034**
2016 vs. 2015	UMBS	5.533	2	.063
2018 vs. 2017	SNWR	35.319	2	**<.001**
2018 vs. 2016	SNWR	61.836	2	**<.001**
2017 vs. 2016	SNWR	26.202	2	**<.001**
2020 vs. 2018	DMP	7.948	2	**.018**
2020 vs. 2017	DMP	6.5761	2	**.037**
2018 vs. 2017	DMP	9.884	2	**.007**
(b) Coyote				
2019 vs. 2018	HMC	4.436	2	.109
2019 vs. 2017	HMC	4.836	2	.891
2019 vs. 2016	HMC	11.043	2	**.004**
2018 vs. 2017	HMC	0.975	2	.614
2018 vs. 2016	HMC	2.543	2	.281
2017 vs. 2016	HMC	3.884	2	.143
2016 vs. 2015	UMBS	5.471	2	.649
2018 vs. 2017	SNWR	0.098	2	.952
2018 vs. 2016	SNWR	1.665	2	.435
2017 vs. 2016	SNWR	1.214	2	.545
2020 vs. 2018	DMP	15.187	2	**<.001**
2020 vs. 2017	DMP	11.27	2	**.004**
2018 vs. 2017	DMP	0.741	2	.690

*Note*: *W* is the test statistic (approximately chi‐sq distributed), and associated degrees of freedom and *p* value are included.

Values were bolded to highlight *p* values that were below the .05 threshold typically used for significance.

Coyote activity was generally more consistent across surveys and years than raccoon temporal activity (Table 2b). Similar to the results for raccoon activity, there was interannual variation in coyote activity on the urban end of the gradient. However, in contrast with the raccoon activity results, this was restricted to only the most urban study area (DMP). The lone exception was the comparison between the HMC 2019 and HMC 2016 surveys, which also showed a significant difference in coyote time use (*W* = 11.043, *p* = .004). This result highlighted a broader trend for coyote temporal activity: there was no significant difference in the temporal activity of coyotes between surveys in a study area unless the surveys were more than a year apart.

### 3.4 Coyote use on raccoon temporal activity

**FIGURE 4:**
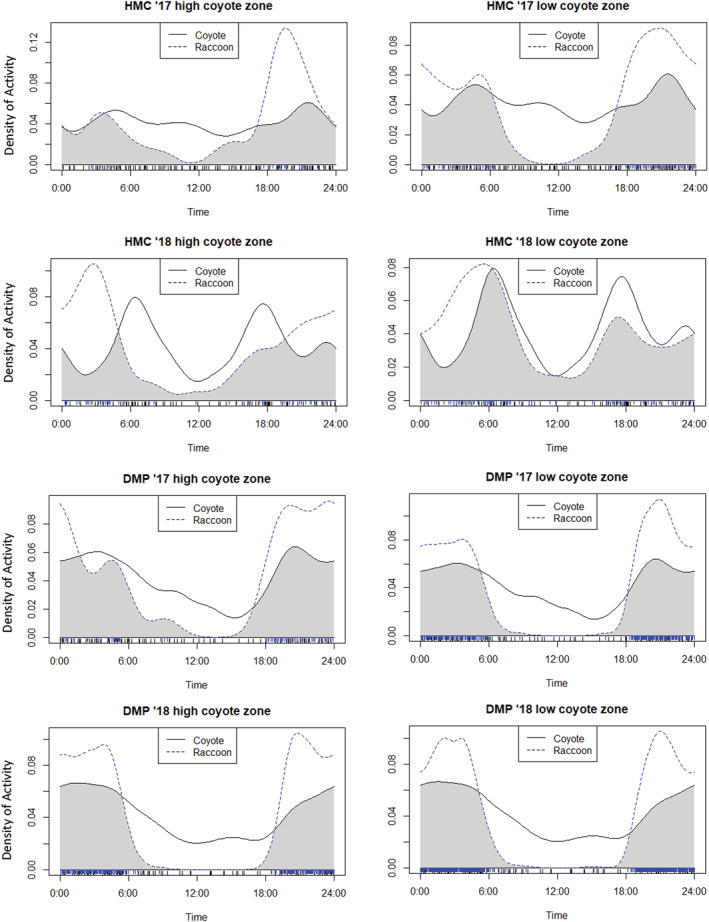
Overlap plots with raccoon temporal activity in high and low coyote zones plotted against aggregated coyote temporal activity for the survey at the opposite ends of the urban–rural gradient for 2017 and 2018 (the 2 years during which both DMP and HMC were surveyed).

Overall, our hypothesis for raccoon‐coyote temporal interactions was largely correct, with raccoons at DMP (the most urban study area) consistently exhibiting no shift in time use relative to coyote intensity of use zones. However, there was reduced overlap between coyotes and raccoons within the high coyote zone. Results for the other areas varied by survey year (Table 3). Below, we first present for each study area the results for the comparison of raccoon activity between the high and low coyote zones. Then we provide the comparison of raccoon and coyote temporal activity within the high coyote zone (relative to the same comparison in the low coyote zone), to determine if there is evidence that a shift in raccoon activity between zones is due to temporal avoidance of coyotes (Figure 4).

**TABLE 3:** Temporal overlap (Δ) coefficients and 95% confidence intervals for raccoon and coyote activity in low and high coyote zones within each camera survey in Michigan from 2016–2020.

Survey period	Site/year	Δ (CI) high	Δ (CI) low	W	*p* value
May–Aug	HMC'19	0.32–0.72	0.28–0.85	10.024	**<.001**
Jun–Aug	HMC'18	0.49–0.76	0.68–0.91	15.122	**.007**
Jul–Jun	HMC'17	0.65–0.83	0.56–0.80	3.841	.147
Jun–Oct	HMC'16	0.59–0.85	0.45–0.83	0.918	.632
Jul–Nov	UMBS'16	0.65–0.85	0.71–0.87	9.631	**.008**
Oct–Dec	UMBS'15	0.42–0.73	0.66–0.86	7.392	**.025**
Sep–Dec	SNWR'18	0.59–0.78	0.66–0.89	10.458	**.005**
May–Aug	SNWR'17	0.60–0.84	0.54–0.88	3.647	.162
Feb–May	SNWR'16	0.63–0.79	0.61–0.81	6.086	**.048**
Jan–Sep	DMP'20	0.46–0.58	0.61–0.80	3.302	.192
Oct–Feb	DMP'18	0.52–0.75	0.61–0.85	0.376	.829
Nov–Mar	DMP'17	0.59–0.83	0.57–0.84	0.692	.708

*Note*: The overlap coefficients for raccoons and coyotes here should be interpreted with caution, as splitting the coyote detections into low and high zones resulted in low numbers of detections in the low zones (and is reflected in the wide confidence intervals). Mardia‐Watson‐wheeler test results comparing raccoon activity between the top quartile and the bottom three quartiles of raccoon activity for each survey are contained in the last two columns, where *W* is the test statistic (approximately chi‐sq distributed), and *p* value are included.

Values were bolded to highlight *p* values that were below the .05 threshold typically used for significance.

**FIGURE 5:**
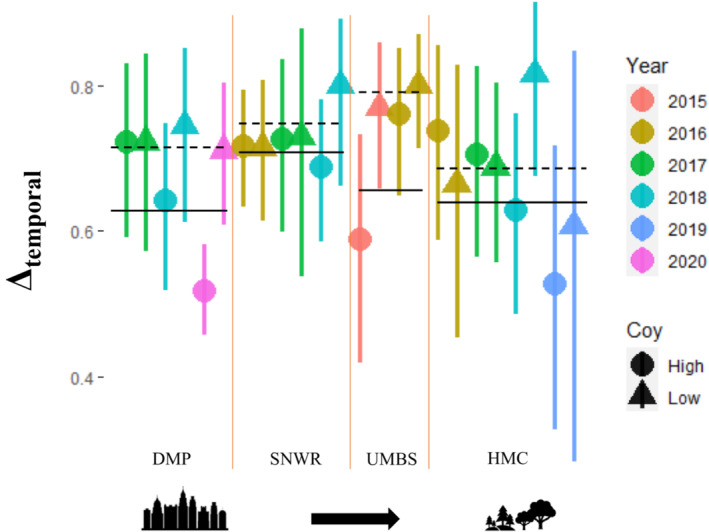
Mean temporal overlap (Δ_temporal_) between raccoons and coyotes in high and low spatial zones of coyote activity with 95% confidence intervals.

HMC: In the most rural study area, we found results for the effects of coyotes varied by the survey. The 2016 and 2017 surveys exhibited no shifts, while surveys in 2018 and 2019 showed significant shifts in raccoon activity between coyote low and high zones (*W* = 15.12, 10.02, *p* < .001, respectively) (Table 3). Results were consistent even when the 2017 survey was separated into summer and winter survey seasons since it covered an entire year, indicating no shifts in raccoon activity between coyote zones. When comparing coyote and raccoon temporal activity within each zone the 2018 survey showed some evidence of decreased temporal overlap between coyotes and raccoons in the high coyote zone, while for 2019 the confidence intervals were too wide to be meaningful (Figure 5).

UMBS: For both surveys, we found there were significant shifts in raccoon activity between coyote zones (*W* = 9.63, *p* < .001 for 2016 and *W* = 7.39, *p* = .025 for 2015). Both surveys also showed evidence of reduced temporal overlap between coyote and raccoons in the high coyote zone.

SNWR: We found that again, results varied by survey, with two out of three surveys showing significant shifts in raccoon activity between coyote zones; 2016 (*W* = 6.08, *p* = .047) and 2018 (W = 10.46, *p* < .001) showed shifts, while in 2017 (W = 3.65, *p* = .162) raccoons did not shift activity. Only the 2018 survey showed evidence of reduced temporal overlap between coyotes and raccoons in the high coyote zone.

DMP: We found that raccoons exhibited no shifts in activity between coyote zones consistently across for all 3 years surveyed in our study. Curiously, two out of the three surveys (2018, 2020) showed evidence of reduced overlap between raccoons and coyotes in the high coyote zone, with the difference reaching significance in the 2020 survey (Δ_4_ CI in the high coyote zone: 0.46–0.58 vs. low coyote zone: 0.61–0.80).

## 4 DISCUSSION

Behavioral adjustments in diet, spatial, and temporal use can reduce competition for resources to promote coexistence (Inouye, 1978). We tested for spatial and interannual variation in the time use of raccoons across an urban–rural gradient and measured the use of temporal refuges by raccoons in the presence of coyotes across that same gradient. As expected, we found that raccoon time use varied both across the gradient and over years. More importantly, we highlight that there were consistent patterns across the urban–rural gradient in raccoon temporal response to coyotes. We found that in the most urban study area (DMP), raccoons consistently did not shift their temporal activity in response to coyotes, despite significant interannual variation in raccoon activity. In contrast, all other study areas showed some evidence of behavioral plasticity in raccoon time use with the intensity of coyote spatial use. Our results lend some support to findings that non‐consumptive or fear effects are present within the hierarchy of the carnivore guild (Gordon et al., 2015), but are overall better explained by human‐associated factors.

### 4.1 Variation in temporal activity across study areas

Urban systems represent an extreme of human pressures, and the continuing increase in urban habitat makes understanding the unique behaviors and ecologies of wildlife in urban spaces such as Detroit, Michigan particularly important. For example, Breck et al. (2019) found that coyotes in urban study areas are bolder in comparison to their rural counterparts. Thus, urban coyotes may be less constrained by the fear of humans in their space and time use. This may explain our surprising result that coyotes were less nocturnal in Detroit compared to the intermediate study areas, the opposite of what we would expect for the avoidance of humans (Gaynor et al., 2018). This temporal activity pattern better fits the similar result from HMC, where the activity pattern we found would be consistent with the avoidance of wolves. Fowler et al. (2021) found little evidence of spatial partitioning between coyotes and wolves in the Upper Peninsula of Michigan (where HMC is located), so temporal partitioning is a plausible coexistence mechanism. The increased nocturnality in the intermediate study areas may be due to the lack of a larger natural predator (the wolf) in combination with higher hunting pressure due to the lower tolerance of rural hunters for coyotes (Drake et al., 2019).

Compared to coyotes, the temporal activity of raccoons seems to consistently become more nocturnal with increasing human pressure. Raccoons have been shown to display a fear response to dog vocalizations in playback experiments, and increasing nocturnality may reflect avoidance of domestic dogs (which are largely diurnal) at the urban end of the gradient (Suraci et al., 2016). Surprisingly, it was not the human‐dominated urban system that was the most unique in raccoon temporal use among the study areas, but instead the more pristine HMC in northern Michigan. The overall raccoon activity pattern showed considerable use of the diurnal period, resulting in low overlap with other study areas. We could similarly attribute this result to the low amount of human and domestic dog presence in the study area.

### 4.2 Interannual variation in temporal activity

HMC was the study area with the greatest interannual variation in raccoon response to coyotes out of the four study areas. One explanation is the lack of human impact in the form of food subsidies, as raccoons rely heavily on anthropogenic foods in some systems (Demeny et al., 2019; Manlick & Pauli, 2020). The availability of resources can modulate the strength of competition, and so annual variation in food resources could drive the avoidance response of raccoons to coyotes (Newsome et al., 2015). In the other three study areas, human food waste and other human‐derived subsidies likely offset years that may otherwise be relatively resource‐poor for raccoons (Oro et al., 2013). Unlike UMBS and SNWR, which have nearby towns, HMC is isolated, surrounded by forest and with the few cabins on the property only seasonally occupied. However, overall raccoon activity (without the consideration of coyote spatial use) showed the greatest interannual variation at the other end of the urban–rural gradient in the two more urban study areas, which would contradict the explanation of anthropogenic food subsidies unless these resources exhibited annual or seasonal differences in the availability. A more plausible explanation is that the level of dependence of raccoons on anthropogenic food subsidies varies based on the season, driving differences in activity patterns. A seasonal dependence on food subsidies would further account for variation between years surveyed in the same fall–winter season (such as DMP'17 and '18), since the onset of cold weather and snow varies annually.

While there was also some interannual variation in coyote temporal activity at DMP, the general consistency in coyote time use from year to year could indicate that coyotes are either less plastic in the temporal niche, are tracking resources and threats spatially, or that there is little variation in resources and threats over seasons and years.

### 4.3 Coyote spatial use on raccoon temporal activity

As the largest wild carnivore in Detroit, the coyote has the potential to act as a fear source for other wild carnivores. In absence of shifts in raccoon activity in our DMP study area, it seems that the fear effect does not extend to raccoons, consistent with the findings of Chitwood et al. (2020). Given that we did find some evidence of temporal avoidance in our other study areas, it is possible that fear of coyotes is not strong enough to elicit a shift in raccoon time use in the face of a stronger force; the most obvious in an urban system being humans and domestic dogs, as reflected by raccoons at DMP having the least diurnal activity (Figure 3a; Gaynor et al., 2018; Nix et al., 2018; Sévêque et al., 2022). Despite raccoon activity consistently being similar between zones of coyote intensity of use, raccoon activity did seem to show somewhat reduced overlap with coyote activity in the high coyote intensity of use areas. This implies that coyotes were potentially using time differently depending on how heavily used an area was by conspecifics. A plausible explanation would be intraspecific competition or aggression (e.g., Newsome et al., 2019), or it could more generally suggest coyotes are more plastic in their time use than raccoons in urban systems (McClennen et al., 2001). The latter is supported by the higher sensitivity of coyotes to human activity; although both species are cosmopolitan, raccoons are more human‐tolerant than coyotes (Crooks, 2002; Green et al., 2022; Randa & Yunger, 2006).

Our results highlight broad patterns in raccoon temporal use between zones of high and low coyote activity. The mechanisms that underlie these patterns require further study and a temporal shift could very likely have more nuance than simple avoidance by a subordinate carnivore. A shift in temporal use by a subordinate (as shown in our SNWR and DMP study areas) might instead reflect the pursuit of an alternate resource (e.g., avoiding exploitative competition by pursuing different prey) rather than avoidance of the antagonistic interaction itself (Newsome et al., 2015). While our results indicate the response of the raccoon to be driven by a larger predator, it does not preclude an interaction between top‐down and bottom‐up forces, which may be important to understanding what raccoons are directly responding to across study areas and survey seasons (Elmhagen & Rushton, 2007). For example, resource availability, such as the abundance of small mammal prey, fluctuates with season and could be a driver of varying levels of competition between coyotes and raccoons (Batzli, 1992; Fedriani et al., 2000; Sovie et al., 2019). Seasonal variation in temporal response may explain the divergent result for the 2017 SNWR survey, which occurred during the summer months. The other two surveys in the study area occurred during the fall and the spring, periods which are associated with heightened resource gathering for the imminent winter, and heightened coyote aggression because of the coyote breeding season (Way et al., 2001). Pairing dietary studies that explore the seasonal variation in coyote and raccoon diets across all study areas with spatiotemporal analyses would elucidate if seasonal variation in resource availability drives resource partitioning between these species.

Though the two study areas at the opposite ends of the gradient (i.e., HMC and DMP) best highlight the variation in raccoon temporal activity and temporal response to coyotes, there were study area‐specific patterns for the entire gradient. We intended for our sampled study areas to represent opposing gradients of humans and native apex predator presence, which were reflected in the number of built structures and which carnivores were captured on camera at each study area. Since we did not explicitly test for the effect of the relative activity of apex predators and humans, we cannot discount the possibility that factors other than top‐down forces drove the urban–rural gradient we observed in our results. Study areas varied in vegetative cover, topography, latitude, and distribution of resources. However, differences in the sources of top‐down forces are the most obvious and likely ecological factor that differs between the study areas for generalist species such as raccoons and coyotes. Similar outcomes have been reported for other coyote‐subordinate predator systems when compared across study areas that vary in the presence of an apex predator (Shores et al., 2019).

### 4.4 Study limitations

The limitations of our study primarily centered around interannual variation and seasonality being confounded. We quantified the interannual variation in raccoon and coyote activity within each study area based on the year of the survey (Table 2). However, since study areas were not surveyed during the same seasons from year to year, the variation that we found could be attributed to either season or year. Furthermore, coyote density and thus activity may fluctuate by year or season. The number of detections of a species is correlated with the trap success, and in our study coyote trap success was fairly consistent within study areas (but see HMC'16 and SNWR'18) (Table S1). When comparing study areas using aggregated raccoon and coyote activity across surveys, the differences in the carnivore community (as mentioned in the methods) in each study area could be a further confounding factor.

## 5 CONCLUSION

We conclude that raccoons may shift their time use in the presence of coyotes. However, it is less clear whether this is done to reduce temporal overlap with coyotes. On the surface, our results seemingly contradict recent works that suggest that coyotes are not an important intraguild predator for raccoons and that raccoons thus do not partition time to avoid coyotes (Chitwood et al., 2020; Gehrt & Clark, 2003). Instead, we suggest that time use shifts may be at a fine scale, and whether they are present depends on a suite of factors. Therefore, for a behaviorally plastic species such as the raccoon, it is difficult to make broad conclusions about time use without considering the variation across the urban–rural gradient they inhabit. Similarly for the coyote, their role as an intraguild aggressor for raccoons is not static across the urban–rural gradient. Instead, the competitive dominance of coyotes is likely dependent on the amount of human pressure and the presence of other larger competitors. Ultimately, as the human footprint on the planet continues to deepen, we need to continually reevaluate interactions across the gradient that it creates. The paradigm in conservation is also shifting to include in‐situ conservation of species in urban habitats, rather than considering these areas solely as suboptimal sink habitats (Athreya et al., 2013; Magle et al., 2012; Mormile & Hill, 2017). Studies comparing the ecological roles of species within a community between urban and natural systems are timely. Such work will prove invaluable in understanding how wildlife communities in these novel habitats differ not just in composition, but also in their function.

## AUTHOR CONTRIBUTIONS

**Rumaan Malhotra:** Conceptualization (lead); data curation (lead); formal analysis (lead); funding acquisition (supporting); investigation (lead); methodology (lead); writing – original draft (lead); writing – review and editing (lead). **Samantha Lima:** Investigation (supporting); writing – review and editing (supporting). **Nyeema C. Harris:** Conceptualization (equal); funding acquisition (lead); investigation (equal); project administration (lead); supervision (lead); writing – review and editing (supporting).

## CONFLICT OF INTEREST

All authors declare that they have no conflict of interest.

## Supporting information


Figure S1

## Data Availability

The datasets used during this study can be accessed at https://doi.org/10.5061/dryad.hx3ffbghc.
