# Supplementary material for: Temporal refuges of a subordinate carnivore vary across rural–urban gradient
Source: Ecol Evol. 2022 Sep 21;12(9):e9310. doi: 10.1002/ece3.9310 (PMC9492470; doi:10.1002/ece3.9310)
Supplement: Supplementary file 1 — Figure S1 [file ECE3-12-e9310-s001.docx]

**Appendix A**

**Fig. S1** Concurrent surveys between study areas used in analysis. At least two surveys were conducted at each study area between 2015 and 2020. Surveys run chronologically from right to left; those surveys that have two survey periods within a year are surveys that were run through December, into the following year (e.g. HMC 2017 ran from July 2017 through May 2018).

**Fig. S2** Example coyote kernel density maps from individual surveys, with camera trap sampling locations from individual surveys.

**Fig.** **S1**


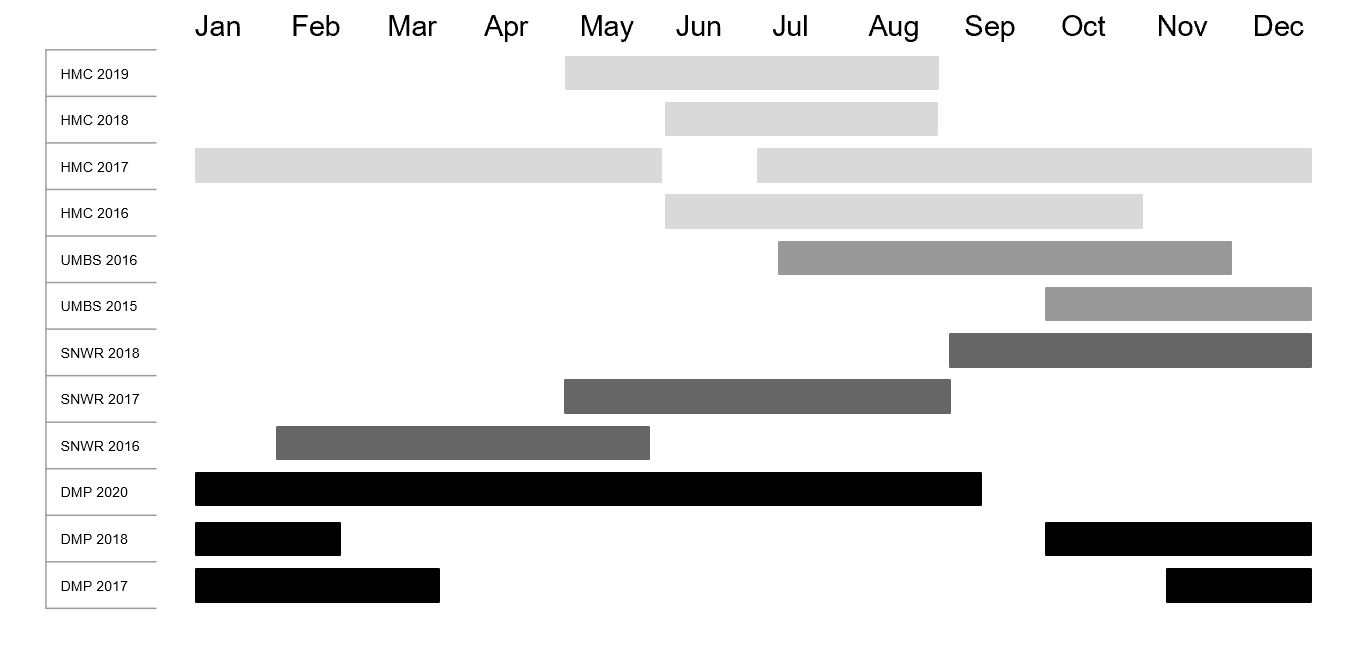


**Fig. S2**

**
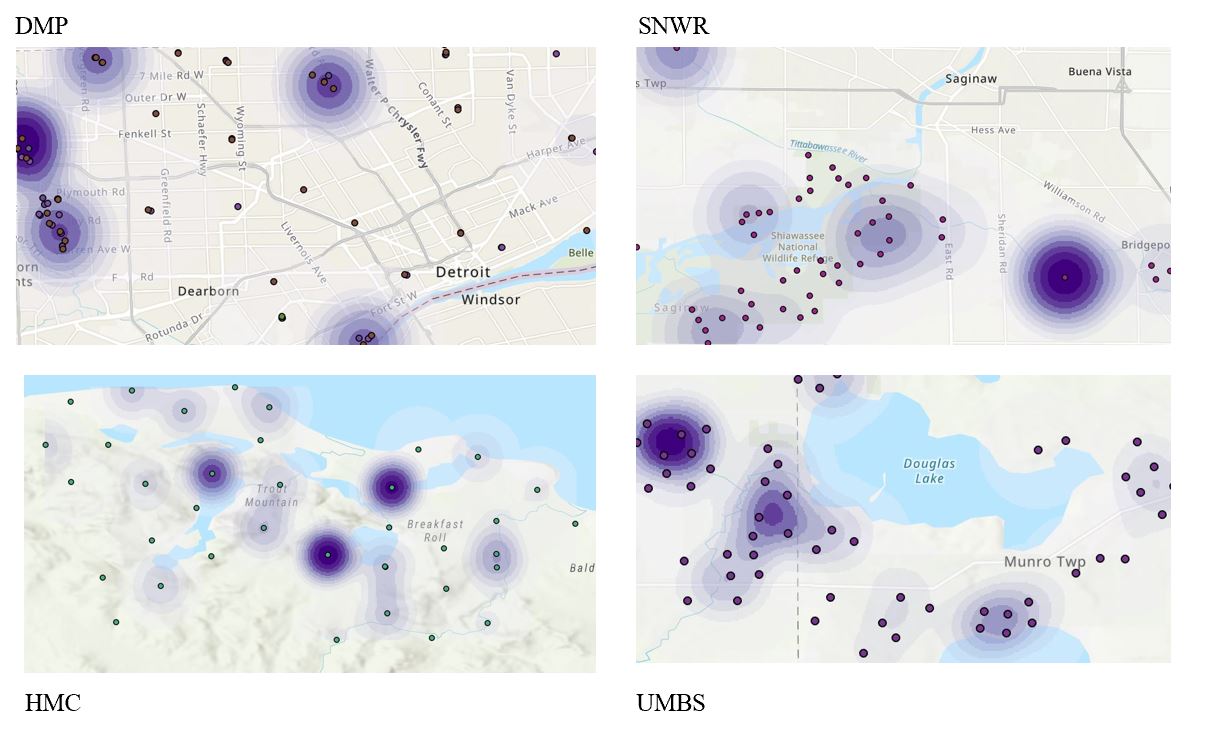
**

**Table S1.** Sampling effort and subsequent detection of the study species for each survey. The number of raccoon and coyote detections are not the number of raw triggers, but instead the number of independent detections after introducing a 30-minute quiet period.

| **Survey period** | **Site/Year** | **Trapnights (n)** | **# Cameras** | **# Raccoon detections** | **# Coyote detections** |
| --- | --- | --- | --- | --- | --- |
| May-Aug | HMC’19 | 3445 | 96 | 70 | 39 |
| Jun-Aug | HMC’18 | 8338 | 43 | 146 | 79 |
| Jul-Jun JJunJunJUNJunJunJun’18 | HMC’17 | 10874 | 43 | 159 | 131 |
| Jun-Oct | HMC’16 | 14211 | 101 | 114 | 48 |
| Jul-Nov | UMBS’16 | 8586 | 61 | 604 | 132  132 |
| Oct-Dec | UMBS’15 | 4367 | 59 | 168 | 89 |
| Sep-Dec | SNWR’18 | 3862 | 41 | 1157 | 48 |
| May-Aug | SNWR’17 | 3076 | 49 | 1175 | 90 |
| Feb-May | SNWR’16 | 5539 | 56 | 2401 | 208 |
| Jan-Sep | DMP’20 | 11713 | 39 | 1635 | 320 |
| Oct-Feb | DMP’18 | 4487 | 41 | 958 | 91 |
| Nov-Mar | DMP’17 | 4097 | 39 | 400 | 97 |
